# In-Vivo Degradation Behavior and Osseointegration of 3D Powder-Printed Calcium Magnesium Phosphate Cement Scaffolds

**DOI:** 10.3390/ma14040946

**Published:** 2021-02-17

**Authors:** Katharina Kowalewicz, Elke Vorndran, Franziska Feichtner, Anja-Christina Waselau, Manuel Brueckner, Andrea Meyer-Lindenberg

**Affiliations:** 1 Clinic for Small Animal Surgery and Reproduction, Ludwig Maximilians University Munich, 80539 Munich, Germany; Katharina.Kowalewicz@chir.vetmed.uni-muenchen.de (K.K.); Franziska.Feichtner@chir.vetmed.uni-muenchen.de (F.F.); a.waselau@lmu.de (A.-C.W.); 2 Department for Functional Materials in Medicine and Dentistry, University of Würzburg, 97070 Würzburg, Germany; elke.vorndran@fmz.uni-wuerzburg.de (E.V.); manuel_brueckner@hotmail.de (M.B.)

**Keywords:** farringtonite, stanfieldite, 3D powder printing, scaffold, biocompatibility, degradable bone substitutes, osseointegration, in-vivo Micro-Computed Tomography

## Abstract

Calcium magnesium phosphate cements (CMPCs) are promising bone substitutes and experience great interest in research. Therefore, in-vivo degradation behavior, osseointegration and biocompatibility of three-dimensional (3D) powder-printed CMPC scaffolds were investigated in the present study. The materials Mg225 (Ca_0.75_Mg_2.25_(PO_4_)_2_) and Mg225d (Mg225 treated with diammonium hydrogen phosphate (DAHP)) were implanted as cylindrical scaffolds (h = 5 mm, Ø = 3.8 mm) in both lateral femoral condyles in rabbits and compared with tricalcium phosphate (TCP). Treatment with DAHP results in the precipitation of struvite, thus reducing pore size and overall porosity and increasing pressure stability. Over 6 weeks, the scaffolds were evaluated clinically, radiologically, with Micro-Computed Tomography (µCT) and histological examinations. All scaffolds showed excellent biocompatibility. X-ray and in-vivo µCT examinations showed a volume decrease and increasing osseointegration over time. Structure loss and volume decrease were most evident in Mg225. Histologically, all scaffolds degraded centripetally and were completely traversed by new bone, in which the remaining scaffold material was embedded. While after 6 weeks, Mg225d and TCP were still visible as a network, only individual particles of Mg225 were present. Based on these results, Mg225 and Mg225d appear to be promising bone substitutes for various loading situations that should be investigated further.

## 1. Introduction

The use of autologous, allogenic or xenogenic grafts is still the gold standard in the surgical treatment of critical size bone defects [1,2,3]. However, they cannot be used for every type of bone defect and involve various risks, such as trauma or infection at the donor site in the case of autografts [4,5], and the transmission of diseases or rejection of the implanted material in the case of allografts and xenografts [6,7]. Because of the many disadvantages of transplants, there has been intensive research in the field of synthetic bone substitutes in recent years [1,8]. The ideal synthetic replacement material is biocompatible and undergoes continuous resorption and, at the same time, is completely replaced by newly formed bone tissue [1,3]. The advantages of synthetic materials are not only their unlimited availability, but also their defined chemical composition and architecture, as well as the possibility to be manufactured to fit any bone defect [9].

Calcium phosphates (CaPs) have become established due to the similarity of their chemical composition to the mineral phase of bone, their excellent biocompatibility and osteoconductive and sometimes even osteoinductive properties [10,11,12,13,14]. In 1920, Albee and Morrison reported the first in-vivo use of a CaP material for bone replacement [15]. Since the 1980s, primary CaPs such as ß-tricalcium phosphate (ß-TCP, Ca_3_(PO_4_)_2_) [10] and hydroxyapatite (HA, Ca_10_(PO_4_)_6_(OH)_2_) [16] have been commercially available as sintered solids, granules or powders, and since the 1990s, also as calcium phosphate cements (CPCs) [17,18]. CPCs are produced by mixing CaP powder with an aqueous solution and are successfully used as bone substitutes in the form of pastes in many fields of application today [19]. However, many CPCs, like most CaP bone substitutes, have the disadvantage of an incomplete and slow degradation from months to years under physiological conditions [20,21,22,23]. The broader clinical application of these cements is also limited by their mechanical properties, since CPCs are brittle, have only low impact strength and relatively low tensile strength [23,24].

In contrast to CPCs, magnesium phosphate cements (MPCs) degrade faster, while showing a more pronounced bone ingrowth due to their higher solubility, and are therefore classified as a suitable alternative bone replacement material [25,26]. Combinations of CPCs and MPCs or the combination of CPCs with magnesium (Mg^2+^) in the form of calcium magnesium phosphate cements (CMPCs) or granulates show even better biological properties than CPCs and MPCs alone, since the chemical solubility, osteoconductivity, degradation rate and resorption of CaPs are increased by the modification with Mg^2+^ ions [27,28,29]. The element Mg^2+^ plays a decisive role in bone metabolism and a lack of Mg^2+^ leads to the loss of trabecular bone mass and increased bone resorption by osteoclasts [30]. The successful use of CMPCs in bone healing has been described in the literature and it has been shown that pastes and pre-hardened cylinders of CMPCs are biocompatible, well-integrated into the surrounding bone and rapidly replaced by newly formed trabecular bone [27,31,32]. However, the use of self-setting cement pastes is limited, e.g., because they are applied into the defect in a viscous condition, have to harden for several minutes at the implant site [33,34,35] and are difficult to shape in some clinical situations [36]. Biocements, which can be produced using three-dimensional (3D) powder printing to form inherently stable, three-dimensional solids of defined shape, offer a more diverse range of possible applications. The variable external structure and macroporosity allow the framework properties to be optimally adjusted to a specific type of tissue that needs to be replaced [37]. 3D powder-printed scaffolds also have a high microporosity (over 30 vol%), which is created by empty spaces between the powder particles [37] and promotes the diffusion of nutrients, vascularization of the scaffold and ingrowth of cells [38,39]. In the past, research has been conducted on such customizable 3D powder-printed scaffolds made of CPCs [40] and MPCs [41]. The aim of the present study was to develop and investigate rapidly degradable CMPC scaffolds using 3D powder printing, as no in-vivo data could be found in the available literature. Two material groups were tested in vitro regarding the influence of a treatment with diammonium hydrogen phosphate (DAHP, (NH_4_)_2_HPO_4_)) on phase composition, pore size and mechanical stability. In vivo, the scaffolds were observed and assessed regarding their degradation behavior and osseointegration in a non-weight-bearing borehole defect in the cancellous part of the lateral femoral condyles in a rabbit model over a period of 6 weeks.

## 2. Materials and Methods

### 2.1. Production of the CMPC Scaffolds

Raw material for the development and production of the cylindrical scaffolds (h = 5.0 mm, Ø = 3.8 mm) was the ceramic cement powder Ca_0.75_Mg_2.25_(PO_4_)_2_, which was produced by sintering (1100 °C, 5 h) 0.5 mol CaHPO_4_ (J.T. Baker, Phillipsburg, NJ, USA), 0.25 mol CaCO_3_ (Merck, Darmstadt, Germany), 1.5 mol MgHPO_4_·3H_2_O (Alfa Aesar, Kandel, Germany) and 0.75 mol Mg(OH)_2_ (VWR, Darmstadt, Germany). Reference implants of the same dimensions were made of tricalcium phosphate (TCP, Ca_3_(PO_4_)_2_) cement. Therefore, a 2:1 molar mixture of CaHPO_4_ and CaCO_3_ was sintered for 5 h at 1350 °C. After manual mechanical crushing of the sinter cakes (grain size approximately 20–30 µm), the powders were homogeneously mixed with the polymeric binder hydroxypropylmethylcellulose (HPMC, 4 wt%, Sigma Aldrich, Taufkirchen, Germany) in a ploughshare mixer (M5R, Lödige, Paderborn, Germany). The scaffolds were then manufactured with a 3D powder printer (ZP310, ZCorp., Burlington, VT, USA) with a layer thickness of 100 µm and a powder-to-liquid ratio of 0.275. The local contact of the HPMC with the printing fluid (degassed water) caused the polymer to swell and thus the ceramic particles to stick together. After drying for 1 h, the scaffolds were removed from the powder bed and freed from adhering powder by compressed air. Subsequently, a multi-stage sintering process with a final sintering temperature of 1100 °C for CMPC or 1350 °C for CPC (heating rate (HR) 1: 120 °C/h, temperature (T) 1: 500 °C, holding time (H) T1: 2 h, HR2: 300 °C/h, T2: 1100 °C/1350 °C, H T2: 4 h) was carried out to burn out the HPMC and compact the ceramic.

The CMPC scaffolds were divided into two material groups. One half of the scaffolds (Mg225) remained untreated after sintering, the other half (Mg225d) received a treatment by immersion (24 h) in an aqueous diammonium hydrogen phosphate solution (3.5 M DAHP, (NH_4_)_2_HPO_4_, Merck KGaA, Darmstadt, Germany). This led to the following chemical reaction:

2 Mg_3_(PO_4_)_2_ + 3 (NH_4_)_2_HPO_4_ + 36 H_2_O → 6 NH_4_MgPO_4_·6 H_2_O + H_3_PO_4_

All scaffolds have been washed to remove residual powder adherence or salt deposits. For this purpose, the scaffolds were stored in a petri dish on a rocking table (Rocker-Shaker PMR-100, Grant bio, Grant Instruments Ltd., Shepreth, UK) and completely covered with 1 mL washing solution per scaffold (sterile distilled water for 1 h, then phosphate-buffered saline (PBS; 8.0 g NaCl, 0.2 g KH_2_PO_4_, 1.1 g Na_2_HPO_4_, 0.2 g KCl in 1 L H_2_O) for 10 min).

Before implantation, all scaffolds were γ-sterilized by BBF Sterilisationsservice GmbH (Kernen, Germany) with a dose of 32.6 kGy.

### 2.2. Phase Determination by X-ray Diffraction and Rietveld Method

Prior to the in-vivo investigation, the phase composition of powders of the printed and subsequently finely ground scaffolds was qualitatively determined with monochromatic radiation (1.541 Å) using the X-ray diffractometer D8 Advance (Bruker Corporations, Billerica, MA, USA). The measurement was carried out in the angular range 2ϴ: 10–50°, with an increment of 0.02°, a measuring speed of 0.5 s/step and with rotation of the measuring cuvette of 15 rpm. The composition of the powder cement samples was evaluated using the software EVA (Bruker Corporations, Billerica, MA, USA) and reference files of the International Centre for Diffraction Data (ICDD) database. The powder diffraction file (PDF) references relevant for this study are summarized in Table 1. In addition, a quantitative analysis of the phase composition via the Rietveld method [42] was performed using the software TOPAS V6 (Bruker Corporations, Billerica, MA, USA).

### 2.3. Determination of Compressive Strength and Porosity

The compressive strength of the scaffolds was measured using a universal testing machine (Z010, Zwick GmbH, Ulm, Germany). The measurement was carried out with a 10 kN load cell at a preload of 1 N and a test speed of 1 mm/min on 10 cuboidal specimens, each measuring 6 mm × 6 mm × 12 mm, which were manufactured in the same way as the cylindrical scaffolds. In approximation to the in-vivo situation, the samples were placed in 30 mL sterile PBS at 37 °C for 24 h prior to measurement. The strength was determined immediately after removal from the medium.

The mercury porosimeter Pascal 140/440 (Thermo Fisher Scientific Inc., Waltham, MA, USA) was used to determine the open porosity, pore size distribution and median pore size of the samples. For this purpose, previously dried (37 °C, 24 h) fragments of the samples from the compressive strength analysis were used. The porosity was measured in a pressure range from 0.01 kPa to 400 MPa. The data was evaluated by the software SOLID (SOLver of Intrusion Data Ver. 1.6.5, Thermo Fisher Scientific Inc., Waltham, MA, USA).

### 2.4. Animal Model

The animal experiment was approved by the regional government of Upper Bavaria in accordance with paragraph 8 of the German Animal Welfare Act (approval number: ROB 55.2-2532.Vet_02-19-64). For this pilot study, the observation period of four female rabbits (Zika rabbits, Asamhof, Kissing, Germany) (age: 6 months, weight: 4.1 ± 0.1 kg) was one (*n* = 1) and six weeks (*n* = 3) post-surgery. The CMPC scaffolds and the tricalcium phosphate (TCP) control group were implanted as shown in Table 2. The animals were kept in standardized single-housing conventional cages (Scanbur A/S, Karlslunde, Denmark). They were fed daily with 100 g pellets and ad libitum hay and water.

Prior to surgery, the animals received enrofloxacin (10 mg/kg, Orniflox^®^, CP-Pharma GmbH, Burgdorf, Germany) and meloxicam (0.3 mg/kg, Melosus^®^, Albrecht GmbH, Aulendorf, Germany) per os (p.o.). Anesthesia was induced by intramuscular (i.m.) application of ketamine (15 mg/kg, Aneskin^®^, Albrecht GmbH, Aulendorf, Germany) and medetomidine (0.25 mg/kg, Dorbene vet^®^, Zoetis Deutschland GmbH, Berlin, Germany). The airways were secured by endotracheal intubation. After shaving and sterile preparation of the surgical site (middle of the thigh to just above the tarsal joint), the animals were placed in a supine position. Anesthesia was maintained by inhalation of isoflurane (1.5–2 vol%, simultaneous supply of oxygen 1 L/min). During the operation, the rabbits received 10 μg/kg/h fentanyl (Fentadon^®^, CP-Pharma GmbH, Burgdorf, Germany) as an intravenous infusion. Buprenorphine (20 μg/kg, i.m., Bupresol^®^, CP-Pharma GmbH, Burgdorf, Germany) was applied close to the end of the surgical procedure for continuing pain treatment.

**Figure 1:**
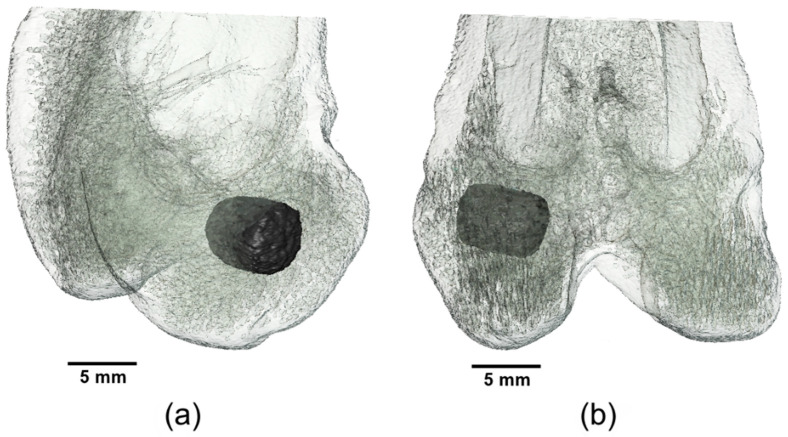
Three-dimensional (3D) reconstruction of the distal rabbit femur with implanted scaffold in the lateral condyle: (**a**) in craniolateral view and (**b**) in caudal view.

The surgical approach was performed through a 5 cm incision of the skin in the area of the left lateral femoral condyle. After preparation of the musculature and exposition of the lateral femoral condyle with aid of a raspatory, a 5 mm deep hole was drilled into the cancellous area of the distal condyle just above the lateral collateral ligament attachment with a drill (Ø = 4 mm) under protection of the joint capsule. The cylindrical scaffold was inserted accurately into the drill hole (Figure 1). Closure of the musculature was performed in three layers (Monosyn 4/0, Braun Melsungen AG, Melsungen, Germany) and the skin was closed with individual sutures (Optilene 4/0, Braun Melsungen AG, Melsungen, Germany). The surgical procedure on the contralateral distal femur was then performed in the same way. Immediately following the operation, X-ray examinations in two views and Micro-Computed Tomography (µCT) examinations of the distal lateral femoral condyles were performed. Medetomidine was antagonized by intramuscular application of atipamezole (25 mg/kg, Atipam^®^, Albrecht GmbH, Aulendorf, Germany). The rabbits were clinically monitored until the anesthesia had subsided completely.

Post-operative follow-up included daily clinical and orthopedic examination of the animals for lameness and pain, as well as wound monitoring until day 14 after surgery. The animals received enrofloxacin once daily (10 mg/kg, p.o.) and meloxicam once daily (0.3 mg/kg, p.o.) for 5 days after surgery. After 10 days, the skin sutures were removed. After the observation periods, the animals were euthanized by intravenous application of propofol (5 mg/kg, Narcofol^®^, CP-Pharma GmbH, Burgdorf, Germany) and pentobarbital (220 mg/kg, Narkodorm^®^, CP-Pharma GmbH, Burgdorf, Germany), in accordance with animal welfare regulations. The scaffold-bone-complexes were explanted using a diamond band saw (cut-grinder Walter Messner GmbH, Oststeinbek, Germany).

#### 2.4.1. X-ray Examination

The hind limbs of the rabbits were X-rayed in two views (ventrodorsal (VD), medio-lateral (ML)) directly after surgery and after 1 respectively 2, 4 and 6 weeks. The images were taken with 54.9 kV and 4.5 mAs (Multix Select DR, Siemens GmbH, Erlangen, Germany). The visibility of the scaffolds in the different X-ray views was assessed using the software dicomPACS^®^ vet (Ver. 8.9.5, Oehm und Rehbein GmbH, Rostock, Germany).

#### 2.4.2. In-Vivo Micro-CT (µCT) Examination

The distal femoral condyles of the rabbits were scanned under general anesthesia directly after surgery and after 1 respectively 2, 4 and 6 weeks in an in-vivo µCT (Xtreme CT II, Scanco Medical, Zurich, Switzerland) with the settings 30.3 µm isotropic voxel size, 68 kV voltage, 1000/180° projections and 200 ms integration time. The animals were placed in supine position with the hind limbs stretched to the maximum.

##### Semi-Quantitative Assessment

The semi-quantitative evaluation of the in-vivo µCT scans was carried out using a specific scoring system. The parameters assessed were scaffold position (time of examination: post-op), scaffold demarcation (spatial and grey scale), scaffold degradation (reduction of diameter and height) and scaffold-bone-contact. Score values of 0–2 were assigned for each parameter (Table 3). For a standardized assessment of the scaffold-bone-contact, rotation of the original scan was necessary to obtain a cross-sectional view of the scaffolds with surrounding cancellous bone [43]. Therefore, the scaffold was manually outlined in the original scan, reoriented and semi-quantitatively evaluated with the software µCT Evaluation Program V6.6 (Scanco Medical, Zurich, Switzerland).

##### Quantitative Assessment

**Figure 2:**
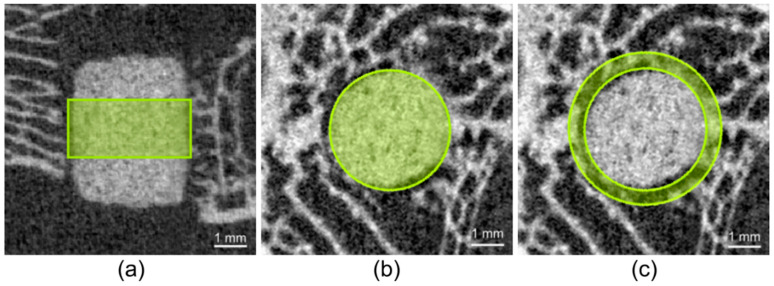
Cylindrical region of interest (ROI) in the scaffold center for measurement of scaffold volume (SV): (**a**) in the original in-vivo µCT scan (Xtreme CT II) (h = 60 slices ≙ 1.82 mm), (**b**) cylindrical ROI in the reoriented in-vivo µCT scan (∅ = 126 voxels (≙ 3.82 mm)), (**c**) second ROI for measurement of bone volume (BV), trabecular number (Tb. N) and trabecular thickness (Tb. Th) in the scaffold environment (inner ring: ∅ = 128 voxels (≙ 3.88 mm), outer ring: ∅ = 160 voxels (≙ 4.85 mm), h = 60 slices ≙ 1.82 mm).

To calculate the volume of the scaffolds and the surrounding cancellous bone in the reoriented scan, it was necessary to define material-specific thresholds (Th). By assessing the grey values of the different scaffolds in the scans directly post-op, the following Ths were determined: Mg225: 144, Mg225d: 149, TCP: 216. The Th for cancellous bone (142) was determined based on the µCT scans of both lateral femoral condyles of cadavers of adult Zika rabbits (*n* = 4) with intact femur bones. To calculate the scaffold volume (SV), a region of interest (ROI) was defined in the scaffold center in accordance with the study by Kleer et al. [43]. The ROI consisted of a cylinder with a diameter of 126 voxels (≙ 3.82 mm), which corresponds to the diameter of the scaffolds, and a height of 60 slices (≙ 1.82 mm = 36.4% of the scaffold height) (Figure 2a,b). The calculation of cancellous bone volume (BV), trabecular number (Tb. N) and trabecular thickness (Tb. Th) around the scaffold was performed according to Diefenbeck et al. [44]. For this purpose, the bone was evaluated in the area with a radius 25% larger than the scaffold. BV, Tb. N and Tb. Th were calculated using a hollow cylinder (second ROI) (inner ring: ∅ = 128 voxels (≙ 3.88 mm), outer ring: ∅ = 160 voxels (≙ 4.85 mm), height: 60 = slices) (Figure 2c). To establish reference values for cancellous bone (BV, Tb. N, Tb. Th), the cancellous part of both distal femoral condyles of cadavers of adult Zika rabbits (*n* = 4) with intact femur bones was examined. All calculations were performed using the software µCT Evaluation Program V6.6 (Scanco medical, Zurich, Switzerland).

#### 2.4.3. Micro-CT (µCT) 100 Examination

The explanted scaffold-bone-complexes were fixed in 4% formalin solution (Roth, Karlsruhe, Germany) and scanned with a µCT 100 at Scanco Medical, Zurich, Switzerland. The samples were fixated in falcon tubes using foam blocks to avoid artefacts caused by movements during scanning. The scans were performed with the settings 8 µm isotropic voxel size, 90 kV voltage, 1000/180° projections and 330 ms integration time. The scans were reoriented and evaluated using the software µCT Evaluation Program V6.6 (Scanco Medical, Zurich, Switzerland).

##### Qualitative Assessment

The qualitative evaluation of the µCT 100 scans was based on both the original scans and the reoriented scans. In each complete scan, bone trabeculae surrounding the scaffold (amount, greyscale, thickness), scaffold integration into the femoral condyle (ingrowth of new bone trabeculae into the scaffold), scaffold degradation and scaffold demarcation of new bone trabeculae (spatially and by grey scale) were assessed, as well as the scaffold structure (cylindrical shape and presence of a material framework).

##### Quantitative Assessment

As described above, the measurement of scaffold volume (SV) in the scaffold center as well as the measurements of cancellous bone volume (BV), trabecular number (Tb. N) and trabecular thickness (Tb. Th) in the scaffold environment were carried out in the reoriented scans in two different ROIs (ROI cylinder for calculation SV in the scaffold center: ∅ = 478 voxels (≙ 3.82 mm), h = 227 slices (≙ 1.82 mm); ROI hollow cylinder for calculation BV, Tb. N and Tb. Th in the scaffold environment: inner ring: ∅ = 485 voxels (≙ 3.88 mm), outer ring: ∅ = 606 voxels (≙ 4.85 mm), h = 227 slices (≙ 1.82 mm)). The determination of the Ths for the different scaffold materials and the surrounding cancellous bone was performed by visual assessment of the µCT scans (Ths: Mg225: 296, Mg225d: 304, TCP: 400, bone: 168).

#### 2.4.4. Histological Examination

After fixation in 4% formalin solution (Roth, Karlsruhe, Germany), the scaffold-bone-complexes were dehydrated in an ascending alcohol series (Roth, Karlsruhe, Germany). The samples were then defatted with xylene (Roth, Karlsruhe, Germany) and embedded with a plastic embedding system based on methyl methacrylate (Technovit 9100^®^, Heraeus Kulzer, Wehrheim, Germany) according to the manufacturer’s instructions. Based on the cutting-grinding technique according to Donath [45], thick sections of 50–80 µm from the center of the scaffold were produced with a diamond band saw (cut-grinder, Walter Messner GmbH, Oststeinbek, Germany) and a grinding machine (lap-grinder, Walter Messner GmbH, Oststeinbek, Germany). The longitudinal axis of the cylinder was perpendicular to the cutting surface of the thick sections. The in-vivo µCT scans were used to determine the scaffold position. A central slide of the sections was routinely stained with a 0.1% toluidine blue O solution [46,47] and evaluated qualitatively. For the preparation of the 0.1% toluidine blue O solution, 1g of Toluidine Blue O (Waldeck, Münster, Germany) was dissolved in 100 mL distilled water to obtain a 1% stock solution. The stock solution was then mixed with a 100 mM phosphate buffer solution at a volume ratio of 1:9 (chemicals from Roth, Karlsruhe, Germany). Before use, the 0.1% toluidine blue O solution was filtered and heated to 60 °C.

##### Qualitative Evaluation

For qualitative evaluation, the histological sections were examined with a microscope (Zeiss Axio Imager Z.2, Carl Zeiss Microscopy GmbH, Jena, Germany). The scaffold area was divided into scaffold center (inner 50%) and marginal area (outer 50%). Scaffold degradation (form and extent of degradation, structure of the scaffolds) and integration into the surrounding cancellous bone, ingrowth of new bone trabeculae, osteoid, connective tissue, blood vessels and bone marrow (magnification 2.5× /0.085) were observed as well as remodeling and resorption processes within the scaffold on the cellular level (appearance of osteoblasts and osteocytes, fibroblasts and fibrocytes, erythrocytes, megakaryocytes, adipocytes, macrophages and giant cells) (magnification 20× /0.5).

### 2.5. Statistics

The examination of the compressive strength of the cuboidal scaffolds was carried out using analysis of variance (ANOVA), followed by a post hoc test evaluated according to Tukey, for a significance level of *p* < 0.05 using the software Origin (OriginPro 2016G, OriginLab, Northampton, MA, USA). The base for the determined phase compositions is formed by three X-ray diffractograms each, which were performed on three scaffold powders. The values are given as mean value ± standard deviation (SD). The clinical, radiological and histological examinations were evaluated descriptively, paying particular attention to the differences between the various materials. The µCT examination was also carried out descriptively (µCT 100), and respectively semi-quantitatively using a scoring system (in-vivo µCT) and quantitatively with the software µCT Evaluation Program V6.6 (Scanco Medical, Zurich, Switzerland) (in-vivo µCT, µCT 100).

## 3. Results

### 3.1. Phase Determination by X-ray Diffraction and Rietveld Method

**Figure 3:**
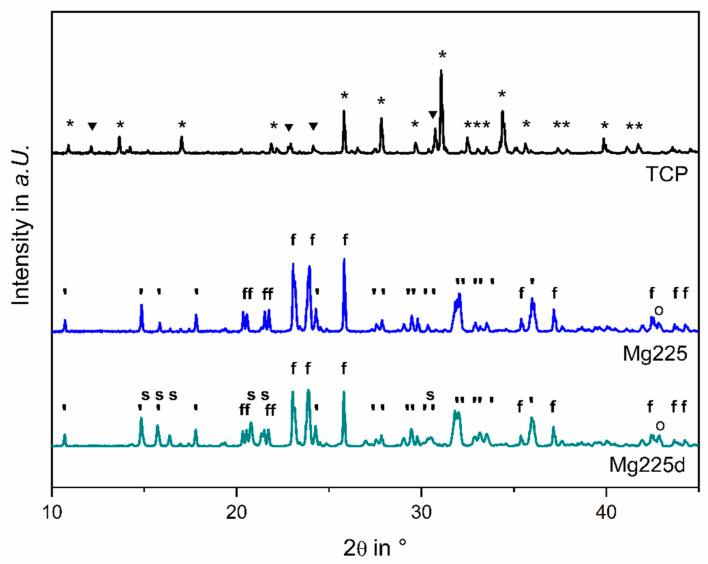
X-Ray diffraction pattern of Mg225, Mg225d and TCP powders. The most relevant peaks are marked for ▼: α-TCP, *: β-TCP, ‘: stanfieldite, f: farringtonite, s: struvite and o: periclase.

The different chemical phase compositions are shown in Figure 3 by the X-ray diffraction patterns of Mg225, Mg225d and TCP measured on powders of the previously finely ground scaffolds. The phase determination via the Rietveld method resulted in the following quantitative material compositions: Mg225 consisted of (60.0 ± 0.4) wt% stanfieldite (Ca_4_Mg_5_(PO_4_)_6_), (38.1 ± 0.5) wt% farringtonite (Mg_3_(PO_4_)_2_), and (1.9 ± 0.0) wt% periclase (MgO). Mg225d consisted of (59.8 ± 0.8) wt% stanfieldite, (31.2 ± 1.1) wt% farringtonite, (6.9 ± 0.3) wt% struvite (MgNH_4_PO_4_·6H_2_O) and (2.1 ± 0.1) wt% periclase. The TCP (Ca_3_(PO_4_)_2_) scaffolds contained (20.4 ± 0.3) wt% α-TCP and (79.6 ± 0.3) wt% ß-TCP.

### 3.2. Determination of Compressive Strength and Porosity

The measured compressive strengths of the different scaffold materials were (4.29 ± 0.77) MPa (Mg225), (10.99 ± 1.68) MPa (Mg225d) and (7.25 ± 1.60) MPa (TCP), respectively. The measurements of the different groups differed significantly from one another (p(Mg225d-Mg225) = 0, p(Mg225-TCP) = 1.96066 × 10^−4^, p(TCP-Mg225d) = 7.28018 × 10^−6^).

**Figure 4:**
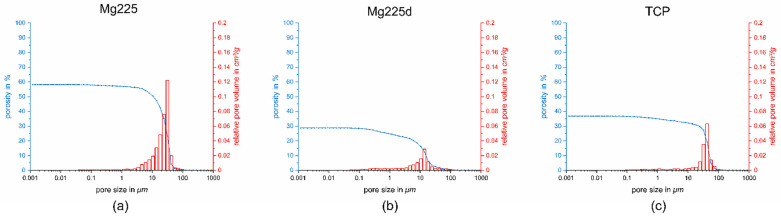
Porosity, pore size and relative pore volume of: (**a**) Mg225, (**b**) Mg225d and (**c**) TCP.

When the porosity was examined using the mercury porosimeter, Mg225 had a porosity of 58%, Mg225d of 29% and TCP of 37%. The mean pore size was 47 µm for Mg225, 13 µm for Mg225d and 30 µm for TCP (Figure 4).

### 3.3. Clinical Examination

As of the first day after surgery, all animals were in good general condition. The rabbits showed no signs of pain, lameness or wound healing disorders. Physiological wound healing took place.

### 3.4. X-ray Examination

**Figure 5:**
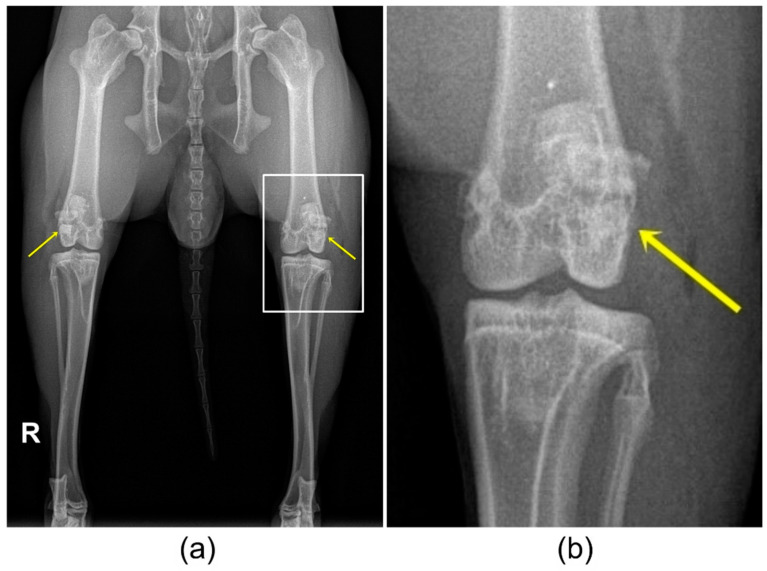
(**a**) Ventrodorsal (VD) X-ray view of the hind limbs directly after surgery with TCP implanted in the right and Mg225 in the left femoral condyle. (**b**) Magnification of the left knee joint with implanted Mg225 scaffold.

The scaffolds had an X-ray density very comparable to that of bone and were visible in the ventrodorsal (VD) image for a shorter time compared to the mediolateral (ML) image. On the X-ray images directly after surgery, all scaffolds were visible in both X-ray views (Figure 5). Over the observation period of 6 weeks, the visibility of the scaffolds decreased differently. Mg225 was only visible in the VD X-ray view directly after surgery, and in the ML view up to week 6. Mg225d was visible in the VD view up to week 4 after surgery, and in the ML view up to week 6. TCP was clearly visible in both X-ray views at all examination times.

### 3.5. In-Vivo Micro-CT (µCT) Examination

#### 3.5.1. Semi-Quantitative Assessment

Except for one sample of Mg225d, which was placed at the transition of the cancellous bone to the medullary cavity of the condyle (score 1), all scaffolds were implanted precisely into the cancellous area of the condyle (score 0).

**Figure 6:**
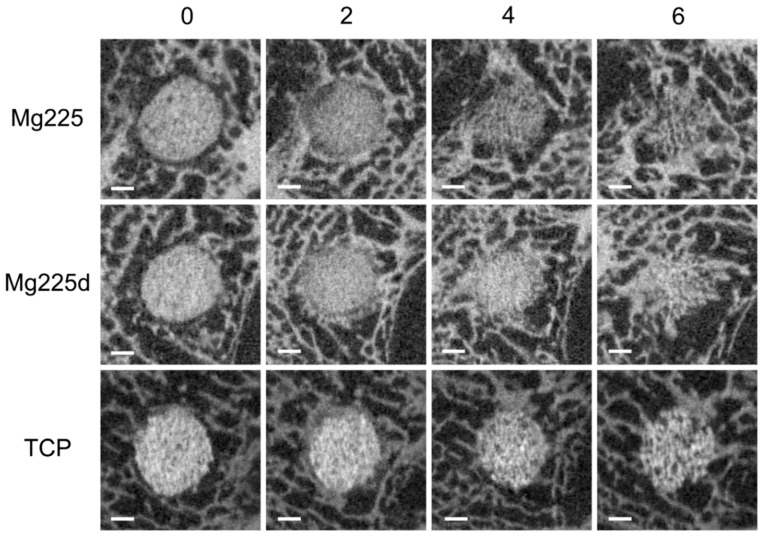
In-vivo µCT images (Xtreme CT II) of the scaffolds and the surrounding cancellous bone in the reoriented scans in cross-section over time (immediately after surgery up to 6 weeks); scale bar = 1 mm.

All scaffolds showed increasing integration into the surrounding bone over the observation period (Figure 6). Until the µCT scan 2 weeks after surgery, all scaffolds were completely demarcable from the surrounding bone tissue (score 2). Four weeks after surgery, both Mg225 scaffolds and one Mg225d scaffold were only partially demarcable (score 1). The second Mg225d scaffold and both TCP scaffolds were still completely demarcable at this time. After 6 weeks, both Mg225 and Mg225d scaffolds, and one TCP scaffold, were only partially demarcable, and the second TCP scaffold was still completely demarcable.

Post-op, no scaffold showed direct contact between scaffold and surrounding bone (score 2). After one week, several bone trabeculae were present between scaffold and surrounding bone in Mg225 (score 1), but not yet in Mg225d (score 2). Two weeks after surgery, the growth of several bone trabeculae was observed in all scaffolds (score 1), except in one TCP scaffold, where numerous bone trabeculae were already present at the scaffold margin (score 0). After 4 and 6 weeks, a wide contact area between scaffold and bone was observed in all scaffolds (score 0).

By week 2, all scaffolds showed no marked reduction in size (h ≥ 4.5 mm, ∅ ≥ 3.5 mm, score 2). In week 4, one Mg225 and Mg225d scaffold each showed dimensions of 2.5 mm < h < 4.5 mm and 2 mm < ∅ < 3.5 mm (score 1), and the other Mg225 and Mg225d scaffold was, as were both TCP scaffolds, still not markedly degraded (score 2). In week 6, all Mg225 and Mg225d scaffolds as well as one TCP scaffold showed dimensions according to score 1, while one TCP scaffold was classified in score 2.

#### 3.5.2. Quantitative Assessment

**Figure 7:**
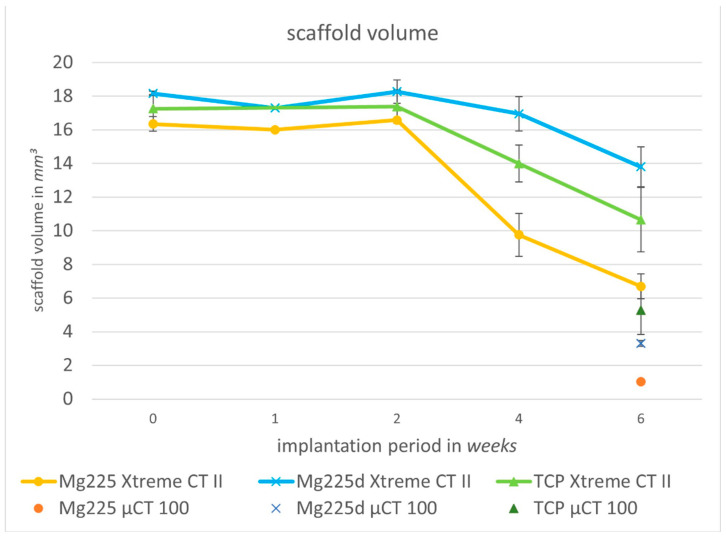
Volume degradation of the scaffolds in in-vivo µCT (Xtreme CT II) over time (immediately after surgery up to 6 weeks) and scaffold volume in µCT 100 (6 weeks after surgery).

All scaffolds showed a continuous volume decrease over the 6-week observation period (Mg225: 59.02%, Mg225d: 23.99%, TCP: 38.24%) (Figure 7). A small increase in the volume of all scaffolds in week 2 post-surgery compared to week 1 and directly post-op was noticeable.

**Figure 8:**
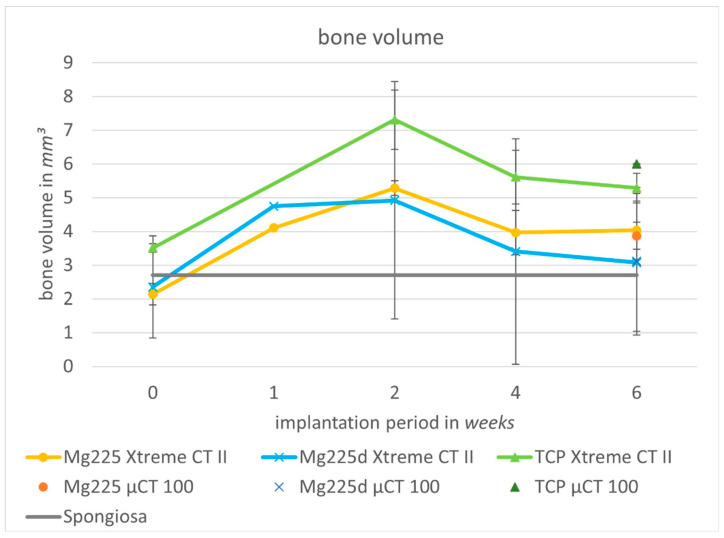
Cancellous bone volume (BV) in the scaffold environment in in-vivo µCT (Xtreme CT II) over time (immediately after surgery up to 6 weeks) and in µCT 100 (6 weeks after surgery) compared to intact cancellous bone of the lateral femoral condyle.

BV increased in all scaffolds when compared directly after surgery with week 6, and from week 1 onwards, the BV was always above the values measured for intact cancellous bone. The highest volume was measured in week 2 (Figure 8).

**Figure 9:**
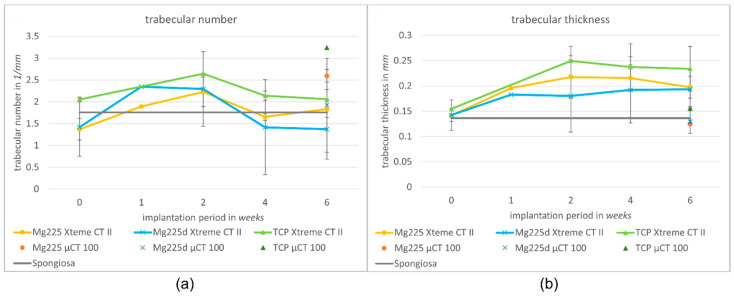
(**a**) Trabecular number and (**b**) trabecular thickness in the scaffold environment in in-vivo µCT (Xtreme CT II) over time (immediately after surgery up to 6 weeks) and in µCT 100 (6 weeks after surgery) compared to intact cancellous bone of the lateral femoral condyle.

When comparing post-op to week 6, Tb. N increased in Mg225 and TCP and decreased slightly in Mg225d. Post-op, all scaffolds showed an increase in Tb. N, with a peak in week 1 (Mg225d) or respectively week 2 (Mg225, TCP). At the following examinations, Tb. N decreased in all scaffolds up to week 6, and respectively increased again slightly in Mg225 between weeks 4 and 6 (Figure 9a).

Tb. Th increased in all scaffolds over the observation period. When comparing the materials, the largest Tb. Th was found in TCP, while the Tb. Th of Mg225 and Mg225d was at about the same level (Figure 9b).

### 3.6. Micro CT (µCT) 100 Examination

#### 3.6.1. Qualitative Assessment

**Figure 10:**
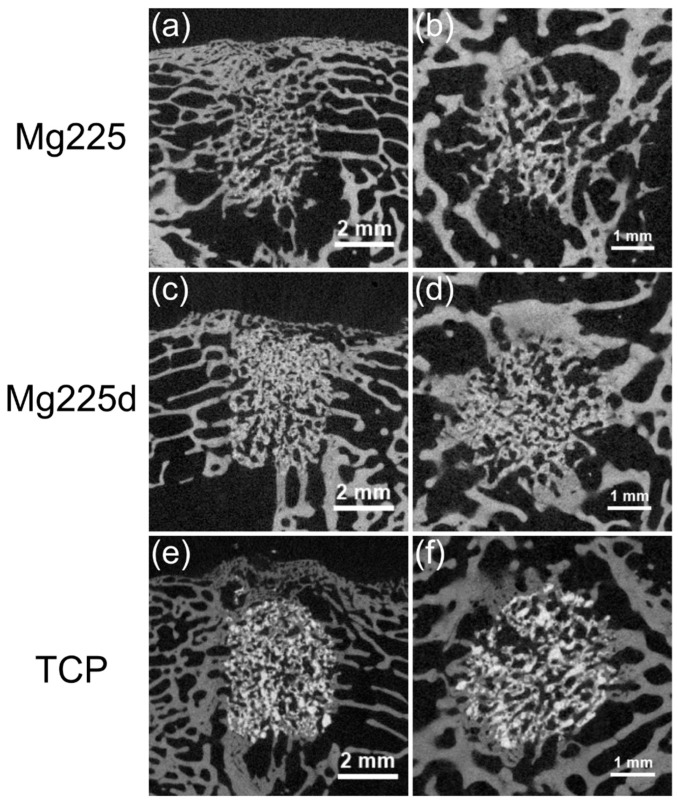
Scaffolds and surrounding cancellous bone (**a**,**c**,**e**) in the original scan in longitudinal-section and (**b**,**d**,**f**) in the reoriented scan in cross-section in µCT 100 6 weeks after surgery.

After 6 weeks, all scaffolds showed a physiological trabecular bone environment. The implantation area was completely traversed by a dense network of new bone trabeculae, which had a slightly lower radiopacity and measurable thickness (Ø = 0.05–0.1 mm) than surrounding bone trabeculae (Ø = 0.1–0.2 mm). The transition between new bone trabeculae within the scaffold and the scaffold surrounding was only partially demarcable, so that the cylindrical shape of the scaffold was no longer visible for Mg225, partially visible for Mg225d and still clearly visible for TCP (Figure 10).

Scaffold residues of Mg225 showed up only as few diffusely scattered single particles (Ø ≤ 0.2 mm) in the initial implantation area, which were completely embedded in new bone trabeculae. A spatial demarcation between bone trabeculae and scaffold was not always clear and was only possible due to the different radiopacity (Figure 10a,b).

The cylindrical shape of the Mg225d scaffolds was relatively clearly delineable. The initial implantation area contained a network of bone and scaffold material. Some individual particles of the material (Ø ≤ 0.2 mm) were scattered in or at the margin of new bone trabeculae (Figure 10c,d).

The scaffold structure of TCP was largely preserved, and the material was clearly distinguishable from bone by means of its grey value. A very dense material skeleton with individual particles scattered in the initial implantation area (Ø ≤ 0.5 mm) was found, some of which were integrated into newly formed bone trabeculae and some of which had no bone contact (Figure 10e,f).

#### 3.6.2. Quantitative Evaluation of µCT 100 Scans and Comparison to In-Vivo Examination

Six weeks after surgery, SVs of 1.02 mm^3^ (Mg225), 3.31 mm^3^ (Mg225d), and 5.28 mm^3^ (TCP) were measured in the µCT 100. The BV was 3.87 mm^3^ for Mg225, 3.12 mm^3^ for Mg225d and 6.00 mm^3^ for TCP. The measured Tb. N was 2.59/mm for Mg225, 1.92/mm for Mg225d and 3.24/mm for TCP, and the Tb. Th was 0.12 mm (Mg225), 0.13 mm (Mg225d) and 0.16 mm (TCP).

When comparing the µCT 100 measurements with the in-vivo µCT measurements of the same time, markedly higher SVs were observed in vivo (Mg225: 6.70 mm^3^, Mg225d: 13.80 mm^3^, TCP: 10.65 mm^3^) (Figure 7), while the BV differed only slightly from the in-vivo measurements (Mg225: 4.03 mm^3^, Mg225d: 3.08 mm^3^, TCP: 5.29 mm^3^) (Figure 8). The Tb. N in the µCT 100 was markedly higher in all scaffolds than in the in-vivo measurements (Mg225: 1.83/mm, Mg225d: 1.37/mm, TCP: 2.06/mm) (Figure 9a), and the Tb. Th was markedly lower than in vivo (Mg225: 0.20 mm, Mg225d: 0.19 mm, TCP: 0.23 mm) (Figure 9b).

### 3.7. Histological Examination

All scaffolds showed a physiological cancellous bone environment consisting of numerous trabeculae and bone marrow at the time of examination 1 and 6 weeks after surgery, respectively. There were no signs of an inflammatory reaction.

**Figure 11:**
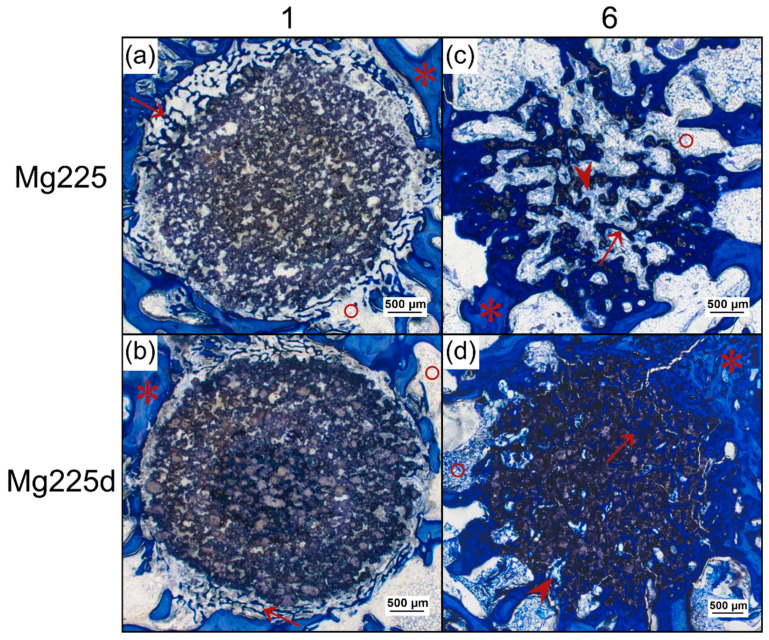
Histological thick sections (toluidine blue staining) from the center of the CMPC (calcium magnesium phosphate cement) scaffolds in cross-section (**a**,**b**) after 1 week and (**c**,**d**) after 6 weeks (magnification 2.5× /0.085). After 1 week, bone trabeculae from the surrounding had grown onto the scaffold and already slightly into the scaffold margin ((*) old bone in lighter blue, (↑) newly formed bone in dark blue, (°) bone marrow). After 6 weeks, new bone trabeculae and (∧) connective tissue traversed the scaffold completely and in the case of (**c**) Mg225, bone marrow had already grown into the scaffold.

One week after surgery, Mg225 and Mg225d showed bone contacts on about 50% of their circumference in the form of numerous new, very thin bone trabeculae, which already grew slightly into the scaffold margin. The scaffolds appeared almost circular in cross-section with a largely homogeneous porous structure. The beginning of degradation was visible by individual unbound scaffold particles in the marginal area. In the entire scaffold area, many cells, especially erythrocytes, were visible between the scaffold particles. In the scaffold margin, connective tissue with many fibroblasts, fibrocytes and lymphocytes had already slightly grown in (Figure 11a,b).

**Figure 12:**
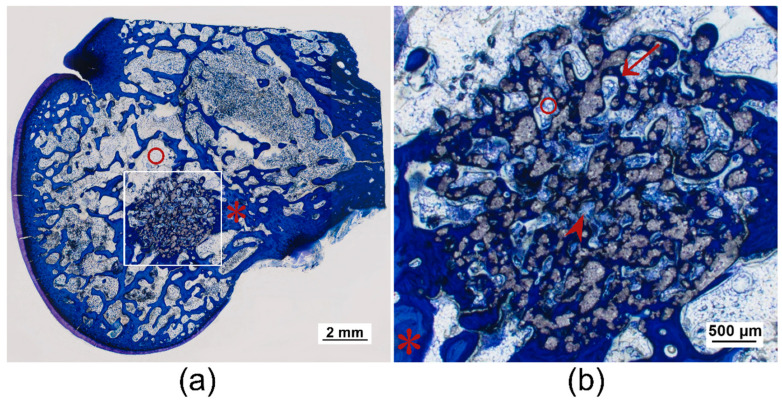
Histological thick sections (toluidine blue staining) of the femoral condyle with TCP scaffold after 6 weeks: (**a**) as overview and (**b**) with magnification 2.5× /0.085. Numerous bone trabeculae from the surrounding had grown into the scaffold and traversed it completely ((*) old bone in lighter blue, (↑) newly formed bone in dark blue), as well as (∧) connective tissue. (°) Bone marrow was also already visible within the scaffold.

After 6 weeks, almost all bone trabeculae from the scaffold surrounding had grown into the scaffold and numerous new bone trabeculae traversed the entire scaffold as a dense network. They appeared somewhat thinner than the trabeculae of the cancellous bone in the scaffold surrounding. At the margins of the many trabeculae within the scaffolds, there was an osteoid layer with numerous osteoblasts deposited on top (Figure 11c,d and Figure 12).

The round cross-sectional area was still relatively easy to identify in TCP and Mg225d, whereas in Mg225, it could only be guessed at. Centripetal degradation occurred in all scaffolds. The Mg225d and TCP scaffolds were visible as a coherent network with some single dispersed particles, which were mainly present at the margins. It was noticeable that the scaffold material was almost always completely surrounded by trabecular bone (Figure 11d and Figure 12). Only single particles of Mg225 were still present, which were embedded in newly formed bone trabeculae (Figure 11c).

**Figure 13:**
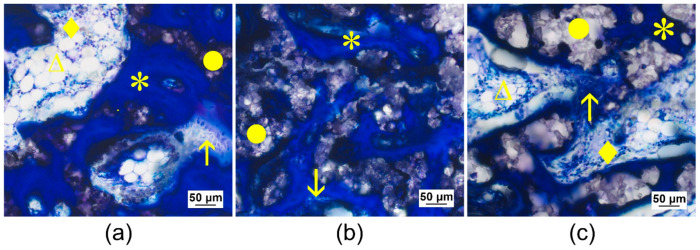
Histological thick sections (toluidine blue staining) from the scaffold center of (**a**) Mg225, (**b**) Mg225d and (**c**) TCP after 6 weeks with magnification 20× /0.5. (•) Remaining scaffold material was surrounded by numerous (*) new bone trabeculae, on the margins of which several (↑) osteoblasts were deposited. In (**a**) Mg225 and (**c**) TCP, (**◊**) cell-rich connective tissue with numerous fibrocytes and (∆) bone marrow (adipocytes with fat vacuoles) were visible between the trabeculae.

In a more detailed examination of the cells inside the scaffold, Mg225 showed homogeneously distributed blood vessels with erythrocytes and lymphocytes in addition to connective tissue (fibroblasts, fibrocytes) and bone trabeculae (osteoblasts, osteoid, osteocytes), which had a comparable quantity and distribution ratio as in cancellous bone outside the scaffold. In the scaffold marginal area, mainly bone marrow with adipocytes, fat vacuoles and megakaryocytes between the bone trabeculae was found, which was also present in numerous smaller locations in the scaffold center (Figure 11c and Figure 13a).

In the case of Mg225d, connective tissue, bone trabeculae and blood vessels with erythrocytes were also observed throughout the whole scaffold. The large number and density of bone trabeculae in the scaffold center was striking. Connective tissue and blood vessels were increasingly located in the scaffold margins. Bone marrow with few adipocytes and megakaryocytes was only sporadically present in the scaffold margin (Figure 11d and Figure 13b).

TCP showed a homogeneous distribution of bone trabeculae and connective tissue throughout the whole scaffold. Bone marrow was present within the whole scaffold area in the form of numerous adipocytes with small fat vacuoles and was surrounded by connective tissue (Figure 12 and Figure 13c).

## 4. Discussion

Due to their better biological properties compared to pure CPCs, CPCs with Mg substitution (CMPCs) are increasingly being researched [27,28,29,35,48] and have proven in various studies to be, in the form of cement pastes, very promising in terms of biocompatibility, osseointegration, successive degradation and absorption [27,28,32,48]. For the treatment of many defects, however, the use of precisely fitting and dimensionally stable bone substitutes of defined shape is necessary. Such materials can be produced using 3D powder printing, but have only been investigated in vivo as CPCs [40] and MPCs [41] so far. In the present study, two different CMPC scaffolds (Mg225, Mg225d) were therefore produced from the ceramic cement powder Ca_0.75_Mg_2.25_(PO_4_)_2_ using the advantageous powder printing process. The influence of the scaffold treatment with DAHP ((NH_4_)_2_HPO_4_) on phase composition, pore size and mechanical stability was tested in vitro. In vivo, the scaffolds were examined in cancellous bone over 6 weeks for differences in degradation behavior, osseointegration, resorption as well as biocompatibility due to the DAHP treatment. They were compared to TCP and evaluated by regular clinical, radiological, µCT, and histological examinations. The rabbit model and chosen implant location has been described as suitable for in-vivo investigations of ceramic bone substitutes [49].

In the present study, treatment of the CMPC scaffolds with DAHP resulted in a change of the chemical composition: Farringtonite (Mg_3_(PO_4_)_2_) (Mg225: (38.1 ± 0.5) wt%, Mg225d: (31.2 ± 1.1) wt%) was partially dissolved by the solution-precipitation process, whereby the in-vivo easily soluble phase struvite (MgNH_4_PO_4_·6H_2_O) (Mg225d: (6.9 ± 0.3) wt%) precipitated and the chemical solubility of the scaffolds was increased. The solubility products of struvite (5.21 × 10^−15^ to 2.12 × 10^−13^ mol^3^ L^−3^ in the pH range 7.01–9.62) [50] and farringtonite (6.3 × 10^−26^ mol^5^ L^−5^) [51], which were reported in the literature, correspond to a calculated solubility of 4.25–14.61 mg/L (struvite) [50] and 2.81 mg/L (farringtonite) [51]. The struvite precipitation resulted in a reduction of porosity and median pore size from 58% and 47 µm (Mg225) to 29% and 13 µm (Mg225d) respectively, and an increase in compressive strength from 4.29 (Mg225) to 10.99 MPa (Mg225d). The reference TCP was between Mg225 and Mg225d in terms of porosity, pore size and compressive strength. The strong increase in compressive strength due to treatment with DAHP was also described by Klammert et al. [52] for 3D powder-printed scaffolds made of MPC and by Gelli et al. [53] for MPC pastes. Gelli et al. [53] also observed a reduction in porosity when MPC pastes were treated with DAHP.

As described for CMPC pastes in other in-vivo studies in a cylindrical defect model in the distal rabbit femur [27,32], the CMPC scaffolds investigated in this study showed excellent biocompatibility without signs of inflammation, rejection or necrosis. In the X-ray examination, the scaffolds had a density comparable to that of bone. In the VD image, the CMPC scaffolds were visible for a shorter time compared to the ML image, as the overlapping of the sesamoid bones in the VD image made it difficult to visualize them. The faster decrease in visibility of Mg225 and Mg225d compared to TCP can be explained by the higher radiopacity of TCP due to the higher content of radiopaque Ca^2+^, but also supports the assumption that the degradation of the CMPC scaffolds happens successively faster than that of TCP. Klammert et al. [54] reported radiological changes in a heterotopic rodent model, which indicates a faster and more distinct dissolution of MPCs compared to CPCs.

In the present study, all three investigated materials showed a continuous, over time increasing scaffold degradation and integration into the surrounding bone in the semi-quantitative (in-vivo) µCT examination. Mg225 was the first material of which both scaffolds could only be partially differentiated from bone tissue after 4 weeks. After one week, growth of bone trabeculae to the margin of the scaffold was already observed in Mg225. After 4 weeks, a wide contact area between scaffold and bone was visible in all materials. In the µCT 100, the cylindrical shape of Mg225 was no longer identifiable after 6 weeks and scaffold residues were only present as diffusely scattered, individual particles, while the outer contour of Mg225d and its material framework were still clearly visible. The scaffold structure of TCP was largely preserved, and the material was clearly distinguishable from bone with the assistance of the grey value. The implantation area of all three materials was completely traversed by a dense network of new bone trabeculae.

For quantitative assessment of scaffold degradation and integration, the SV and its surrounding BV, Tb. N and Tb. Th were measured. Until week 2, the SV remained more or less constant for all materials, and even a slight increase in volume was observed, which could not be clearly explained in this study. After the implantation of a biodegradable material, protein adsorption and subsequent cellular infiltration, especially of monocytes, macrophages and foreign body cells, occur very rapidly. One to five days later, these cells release cytokines and chemokines, resulting in the recruitment of tissue repair cells after five to fifteen days [55]. It is therefore assumed that in the present study, hardly any cellular resorption processes of the scaffold material were present in the first two weeks after surgery, as the required signal transduction cascade had to proceed first. However, it can be assumed that a slight volume decrease by chemical dissolution occurred in all materials even before week 2. Histology showed that cells and especially new bone trabeculae had already grown into the slightly degraded scaffold after 1 week and were erroneously included in the SV due to the very similar grey value of bone and scaffold material in µCT. From week 2 onwards, a severe decrease in the scaffold volume was observed in all materials, presumably due to the increasing cellular resorption of the scaffolds. This was more pronounced than the chemical dissolution that probably already occurred previously and was clearly measurable despite the further ingrowth of cells and new bone trabeculae. Mg225 showed a markedly stronger and faster volume decrease than Mg225d and TCP (volume decrease over 6 weeks: Mg225: 59.02%, Mg225d: 23.99%, TCP: 38.24%). The specific degradation mechanisms of the scaffolds investigated in the present study could not be clarified conclusively. However, the degradation of CaP-based biomaterials has been investigated in many studies and chemical dissolution and cell-mediated absorption have been assumed [56,57,58]. In-vitro studies on the degradation of brushite cements have shown that degradation occurs through erosion, fragmentation and dissolution, with calcium and phosphate ions diffusing in solution [59]. For the degradation of CPCs in vivo [24], the same degradation mechanism is assumed as for CaP ceramics [60], although the cell type involved in the degradation varies depending on the cement type. Rapidly resorbable CPCs are removed by macrophages and giant cells that phagocytize cement particles [57,58,61], while CPCs, which are resorbed more slowly over months to years, are removed by osteoclastic cells [21,57]. For MPCs, only passive resorption by chemical dissolution in magnesium and phosphate ions has been described in the literature so far [54,62]. For the in-vivo degradation of pre-hardened cylinders made of CMPC pastes, Wu et al. [27] have assumed a two-stage degradation mechanism with chemical dissolution and resorption of the material at the initial stage and cell-mediated resorption later on, which we also consider likely for the scaffolds examined in the present study. Both the surface quality and the chemical composition of a biomaterial are decisive for cellular reactions to it [63,64]. In the present study, despite the higher proportion of the chemically poorly soluble phase farringtonite in Mg225, the larger pores and higher porosity resulted in better osseointegration and markedly faster resorption of the Mg225 scaffold compared to Mg225d. The influence of pore size and porosity on the degradation rate has been described in other studies, showing that high porosity and large pores facilitate and improve osteogenesis and osseointegration of the scaffold, as they allow vascularization of the construct and a high oxygen supply [39,65,66]. However, an increase in pore size reduces the mechanical strength of the scaffold and, as bone grows in, the mechanical properties of the scaffold are further reduced by impairment of the framework’s structural integrity, which can be critical for regeneration in weight-bearing bone [39]. In addition to the compressive strength of a bone replacement material, however, its stiffness (compressive modulus) and the right combination of both mechanical properties play a decisive role in its use to fulfill the mechanical functions of the bone matrix [67]. As compressive strength and stiffness are negatively correlated to the porosity of a material [68,69], it can be assumed that the Mg225 scaffolds have a lower stiffness than the Mg225d and TCP scaffolds, at least prior to implantation. This will possibly change during implantation time in vivo, as the larger pores and porosity of Mg225 facilitate the ingrowth of cells [39,65,66], which has been shown to have an impact on the scaffolds’ stiffness [70] and should therefore be taken into account to be evaluated in possible follow-up studies on CMPC scaffolds. Liverani et al. [70] have investigated the influence of cell colonization on the stiffness properties of collagen scaffolds and observed an increase in the scaffold compressive modulus. Although Mg225d showed less volume degradation than Mg225 and TCP by week 6, an increasing and complete degradation of both CMPC scaffolds over time is nevertheless likely according to the existing literature. Li et al. [71] investigated CMPC scaffolds made of pastes in vivo in the distal rabbit femur and observed a complete degradation of the material and regeneration of bone tissue over 6 months, with the largest bone growth observed between months 1 and 3. The fact that after 6 months no scaffold material could be detected allows the conclusion that possible follow-up studies of the present pilot study do not need to extend beyond 6 months.

In the scaffold environment, a clear increase in BV was observed when comparing directly after surgery to week 6, which was most pronounced in the untreated CMPC (Mg225). Strikingly, the BV was highest for all materials in week 2, and from week 1 onwards, it was always higher than the BV of intact bone of the same localization. As the outer contour of the scaffolds appeared blurry in the in-vivo µCT already from week 1, it is assumed that the chemical dissolution of the scaffolds occurred mainly in the early implantation phase and had a positive influence on the formation of new bone trabeculae. The amount of free Ca^2+^ and PO_4_^3−^ ions is known to have a crucial effect on bone metabolism. A local increase of Ca^2+^ and PO_4_^3−^ ions to supra-physiological levels released by osteoclasts during in-vivo bone resorption severely affects the proliferation and differentiation of osteoblasts and the following bone formation [72]. A high Ca^2+^ concentration stimulates the chemotaxis of pre-osteoblasts to bone resorption areas as well as their maturation into bone-producing cells [73], while PO_4_^3−^ plays an important role in the regulation of cell cycles and proliferation rates [74]. In the present study, the release of Ca^2+^ and PO_4_^3−^ ions during scaffold degradation likely led to a local increase of the Ca^2+^ and PO_4_^3−^ concentration in the scaffold environment, especially in the early implantation phase. New bone formation was probably stimulated thereby, resulting in a higher BV in the scaffold environment compared to intact cancellous bone. This would also explain the higher BV in the environment of the TCP scaffolds compared to the CMPC scaffolds as they contain a markedly larger amount of Ca^2+^. In addition, the diffusion of scaffold material into the environment probably led to changes in the microstructure of the scaffolds, which could facilitate the presumably later occurring cell-mediated resorption of the CPC phase and has been suggested in the literature for the degradation of pre-hardened cylinders made of CMPC pastes [27]. However, a stimulation of bone formation by the MPC phases of the scaffolds is also conceivable as an explanation for the high BV. Since Mg^2+^ promotes mesenchymal stem cell differentiation into osteoblasts and osteoblast activity [75,76,77], it is possible that Mg^2+^ released during the chemical dissolution of the CMPC scaffolds promoted osteoneogenesis. Kanter et al. [78] also found a markedly higher BV in the vicinity of struvite cements compared to intact bone from the same region, which suggests that struvite cements stimulate bone formation, possibly through the Mg^2+^ ions released. In a previous study by Ewald et al. [32], Mg225d cement pastes were examined in a rabbit model and an increase in scaffold-bone-contacts from 6 to 12 weeks was observed. This suggests that for the 3D powder-printed scaffolds of the present study, an increase in osseointegration over time is also to be expected, although this must be clarified in subsequent studies.

Tb. Th increased for all materials over the investigation period, while by comparison of post-op with week 6, Tb. N increased for Mg225 and TCP and decreased slightly for Mg225d. An increase in Tb. Th and decrease in Tb. N was also described by Kanter et al. [78] for struvite cements and indicates that the new bone is continuously remodeled and adapted to the physiological situation.

Since the CMPC scaffolds in the present study had a very similar grey level as surrounding bone, the quantitative in-vivo µCT investigations were validated with additional examinations of the explanted scaffold-bone-complexes in a µCT 100 at the same time point (6 weeks). Marked differences in the measurements of degradation and osseointegration parameters were found. While all scaffolds showed a lower SV and Tb. Th in the µCT 100, the Tb. N was higher than in the lower-resolution in-vivo µCT examination. The BV in the scaffold environment was comparable in both measurements. The discrepancy in measurements can be explained by the smaller isotropic voxel size (8 µm compared to 30.3 µm) and the resulting higher accuracy of the µCT 100 measurements. New bone trabeculae in the cylindrical ROI in the scaffold center can be better separated from the scaffold material due to the more differentiated grey scales, and bone trabeculae in the scaffold environment can be detected individually in a more differentiated manner. These observations can also be found in other studies on trabecular microstructure parameters, which also concluded that small trabeculae are poorly represented by low-resolution systems [79,80]. However, since measurements in the µCT 100 can only be made on the explanted scaffold-bone-complexes after euthanasia of the animals due to the smaller scan range, the in-vivo investigation is nevertheless indispensable for assessing degradation and osseointegration over time.

Histological examinations confirmed the observations from the µCT investigations regarding scaffold degradation and integration. By increasing implantation time, both CMPC scaffolds induced the formation of new bone trabeculae. Contrary to the assumption that a minimum mean pore size of 100 µm is required for the regeneration of mineralized bone tissue within a scaffold in ceramic bone substitutes [81], in the present study, new trabeculae had slightly grown into the margin of scaffolds with a median pore size of 13 to 47 µm already, one week after implantation, and completely traversed the scaffolds of all materials as a dense network after 6 weeks. An osteoid layer with numerous deposited osteoblasts was present at the trabecular meshwork margins. In the case of Mg225 and TCP, cell-rich tissue was already visible, particularly at the margin of the scaffolds, which was evaluated as bone marrow due to the numerous occurrence of adipocytes, erythrocytes and megakaryocytes [82]. Increased bone growth was accompanied by vascularization, centripetal volume degradation and structural loss of the scaffolds, which was also observed in other in-vivo studies [31,32,62]. The scaffold material was almost always completely surrounded by trabecular bone, but while Mg225d and TCP were still visible as a coherent grid network, only single particles of Mg225 were still present. The integration of material particles in new bone was also observed by Kim et al. [62] in a previous in-vivo study with MPCs and suggests that the bone has newly formed, remodeled and matured. As reported in the literature [27], the clearly visible osseous remodeling processes in the implantation area and the centripetal scaffold degradation seen in the present study indicate a partial cell-mediated dissolution of the scaffolds. However, it was striking that no giant cells or macrophages were observed in the present study.

The higher porosity and pore size of Mg225 compared to Mg225d and TCP provoked faster vascularization, which was also observed for MPC scaffolds of different micropore sizes (25–53 µm compared to <25 µm) in another study [62]. A correlation between the pore structure of ceramic scaffolds and their efficiency in bone formation and remodeling was also described [62]. The faster migration of mesenchymal cells and osteoblasts [65,66] into the scaffold finally led to a faster dissolution and osseointegration of the Mg225 scaffold compared to the Mg225d and TCP scaffolds in this study.

The importance of the exact positioning of the scaffold for successful osseointegration is demonstrated by one Mg225b scaffold, which was not inserted exactly into the cancellous part of the condyle, but at the transition of cancellous bone to the medullary cavity. Since it was thus not completely surrounded by bone trabeculae, poorer osseointegration occurred and BV, Tb. N and Tb. Th were markedly lower than with the other Mg225d scaffold during the whole observation period. Furthermore, other studies described implant site-dependent degradation behavior of magnesium-based implants, revealing that scaffolds implanted in cancellous bone close to bone marrow or in the bone marrow degrade faster than in cancellous or compact bone [83,84,85]. However, no faster degradation of the scaffold implanted at the transition of cancellous bone to the medullary cavity compared to the scaffold implanted in cancellous bone was observed in the present study.

## 5. Conclusions

The treatment of the scaffold material Mg225 with DAHP, which was investigated in the present pilot study, led to a change of the chemical phase composition and thus to a reduction of pore size and porosity and an increase in compressive strength in Mg225d. The 3D powder-printed materials showed excellent clinical tolerance, biocompatibility and biodegradability, with no signs of inflammation. Degradation with loss of shape and structure, volume decrease as well as the decreasing differentiability from bone tissue was more pronounced and faster in Mg225 than in Mg225d and TCP. The scaffolds showed rapid degradation, good osseointegration and effective osteogenesis, as numerous new bone trabeculae and, in the case of Mg225 and TCP, bone marrow had grown into the scaffolds. As the mechanical stability of the scaffold-bone-complexes in vivo has not been investigated, only a report on the in-vitro stability of the scaffolds is possible at this time. The relatively high porosity of Mg225 and associated low compressive strength promote osteogenesis and osseointegration but make this material unsuitable for use in weight-bearing bone. However, Mg225 is probably well-suited as a substitute for cancellous bone in cavities. Based on the results from the present study, Mg225d seems to be a promising bone substitute for the use in complex shaped or more weight-bearing defects. The longer lasting, coherent gridwork serves as a guide for osteogenesis, which could lead to an increase in mechanical stability in the implantation area and needs to be further investigated in follow-up studies.

## Figures and Tables

**Table 1:** Summary of powder diffraction file (PDF) references relevant for this study.

Phase	PDF-Reference
α-TCP (Ca_3_(PO_4_)_2_)	06-0200
β-TCP (Ca_3_(PO_4_)_2_)	09-0169
Farringtonite (Mg_3_(PO_4_)_2_)	33-0876
Periclase (MgO)	04-0829
Stanfieldite (Ca_4_Mg_5_(PO_4_)_2_)	11-0231
Struvite (NH_4_Mg(PO_4_)·6 H_2_O)	15-0762

**Table 2:** Combinations of implanted materials.

Animal Number	Scaffold Type (Left Lateral Condyle)	Scaffold Type (Right Lateral Condyle)	Time of Euthanasia in Weeks after Surgery
1	Mg225	Mg225d	1
2	Mg225d	Mg225	6
3	Mg225d	TCP	6
4	Mg225	TCP	6

**Table 3:** Scoresheet for the semi-quantitative evaluation of the in-vivo Micro-Computed Tomography (µCT) (Xtreme CT II) scans.

Parameter	Score 0	Score 1	Score 2
Scaffold position	Scaffold completely inside the cancellous condyle	Scaffold mostly (>50%) in the cancellous condyle, partially in the medullary cavity	Scaffold partially in the cancellous condyle, mostly (>50%) in the medullary cavity
Scaffold demarcability	Scaffold cannot be separated from surrounding bone tissue	Scaffold partially separable from surrounding bone tissue	Scaffold can be completely separated from surrounding bone tissue
Scaffold degradation	Scaffold size	Scaffold size	Scaffold size
	h ≤ 2.5 mm	2.5 mm < h < 4.5 mm	h ≥ 4.5 mm
	∅ ≤ 2 mm	2 mm < ∅ < 3.5 mm	∅ ≥ 3.5 mm
Scaffold-bone-contact	Broad contact area between scaffold and surrounding bone, numerous trabeculae grown onto the scaffold, no gap visible	Several bone trabeculae between scaffold and surrounding bone, hardly visible gap	No contact between scaffold and surrounding bone, clear gap between bone and scaffold

## Data Availability

The data presented in this study are available on request from the corresponding author.
